# Mental suffering and the Brazilian National Health System

**DOI:** 10.1590/S2237-96222023000100001

**Published:** 2023-03-27

**Affiliations:** 1 Universidade Estadual de Campinas, Faculdade de Ciências Farmacêuticas, Campinas, SP, Brazil

This first issue of Epidemiology and Health Services: journal of the Brazilian National Health System (*Epidemiologia e Serviços de Saúde: revista do SUS* - RESS) in 2023 brings six relevant contributions regarding mental health. The challenges to building policies for mental health care and work-related distress are discussed, shedding light on the existing scenario and perspectives in this area.^1^ The mental health of primary care workers was investigated in a survey conducted in Minas Gerais in 2021, looking at effects of work overload and previous disorders on the occurrence of mental illness during the COVID-19 pandemic.^2^ Two analyses based on data from the Hospital Information System found a reduction in psychiatric hospitalizations in the Brazilian National Health System *(Sistema Único de Saúde* - SUS) between 2008 and 2021,^3^ and hospitalizations due to alcohol use between 2010 and 2020,^4^ possibly reflecting effects of the psychiatric reform and its fight anti-asylum, as well as consequences of the pandemic on the chain of care. An extreme indicator of mental suffering, suicide, was assessed using data from the Mortality Information System for the period 1996-2018 and found higher risk among male and the elderly in Chapecó/SC, as well as identifying geographical regions at greater risk based on spatial analysis.^5^ A cohort based on High Complexity Procedure Authorizations for the period 2008-2017, found that off-label use of atypical antipsychotic medications in children and adolescents was frequent among health service users seen by the Specialized Pharmaceutical Care Component of the SUS.^6^

A point common to the studies mentioned above was that data generated within the SUS were the source of these original articles. Four of these studies were based on systems comprised of data input by health service workers and managers,^3^ ^-^ ^6^ highlighting the role of the SUS in providing information that is the basis for research that provides feedback to the health system and society, in addition to providing comprehensive and universal care in its widespread network. The recording of and access to information originated in the various activities of the SUS, and its improvement through indicator analysis, must be recognized and encouraged throughout society, especially by the scientific community.

All these human activities, both care and managerial, are performed by SUS workers, who also need to be valued. According to the Minas Gerais survey, primary care professionals suffered the consequences of mental health overload, which also indicates the role played by health services in the illness of people who work in them.^2^ In addition to the stress resulting from work activities, threats to the job stability of health workers, posed by outsourcing and precarious labor relationships, have become frequent in the context of the SUS and need to be combated to prevent mental illness among health workers.^1^

There is a close relationship between mental suffering and social determinants. Feelings of uneasiness, such as worry, fear, shame, loss of identity and belonging, perception of lack of control in face of financial difficulties - commonly found in situations of unemployment, for example - underlie the biological rationale between context and mental suffering that lead to both individual and community illness.^7^ ^,^ ^8^ In a contradictory manner, the neoliberal viewpoint seeks to pulverize collective problems into fragmented issues. Failures would be the fault of individuals or families, seen as undertakings that require adequate and responsible management that does not burden the State. From this meritocratic perspective, weakened society becomes overshadowed, and discussions to resolve historical injustices are rendered impossible. Health problems are particular issues to be dealt individually to ensure productivity. Likewise, mental suffering is a dysfunction to be solved individually, normalizing medicalization of life. The very access to health care is a personal problem that must be solved according to one’s economic condition - ultimately a reflection of previous choices - legitimizing universal health coverage, in which everyone pays according to their means, to the detriment of the universal health system based on solidarity, such as the SUS. It is evident that there is suffering when concern about socioeconomic issues is absent. Such distress is a reflection of human nature involving feeling and maturing permeated by emotions, and which can result in mental disorders that require appropriate therapy. Far from depersonalizing individuals by reducing them to their contexts, the relationship between mental health and society is inseparable.

The RESS reinforces its role as a promoter and disseminator of research that strengthens the SUS, in its different aspects and providing visibility to essential themes for Brazilian society, based on the understanding that the population’s health - especially mental suffering - is not restricted to a medical issue.
